# Menopausal status, age at menopause and risk of all-cause mortality among Chinese women: findings from a 10-year prospective study

**DOI:** 10.1136/bmjph-2023-000332

**Published:** 2023-12-22

**Authors:** Sha Huang, Ruofan Gongye, Siyu Zou, Jia Yi Hee, Kun Tang

**Affiliations:** 1 School of Life and Health Sciences, Hainan University, Haikou, China; 2 Vanke School of Public Health, Tsinghua University, Beijing, China

**Keywords:** Public Health, Epidemiology, Female

## Abstract

**Introduction:**

Menopause characteristics (menopausal status and age at menopause) have been implicated in future health consequences. However, evidence of the impact on menopause on total mortality has been inconsistent. The present study aimed to investigate the associations of menopausal status and age at menopause with the risk of all-cause mortality in Chinese women.

**Methods:**

We used prospective data from the China Kadoorie Biobank cohort study that recruited over 300 000 women aged 30–79 years from 10 regions across China between 2004 and 2008. All participants were prospectively followed up, with a median follow-up of 10.20 years. Cox proportional hazard regression was used to examine HRs with 95% CIs for all-cause mortality associated with menopausal status and age at menopause.

**Results:**

Compared with premenopausal women, postmenopausal women were at higher risk of all-cause mortality with adjusted HR of 1.11 (95% CI 1.03 to 1.20). Among postmenopausal women, the HRs of total mortality were 1.25 (95% CI 1.14 to 1.36), 1.09 (95% CI 1.03 to 1.15), 0.98 (95% CI 0.94 to 1.02) and 0.97 (95% CI 0.91 to 1.04) for menopause at ages less than 40, 40–44, 50–53 and 54 years or older, respectively, relative to 45–49 years. In addition, for each 1-year increase in age at menopause was associated with a 1% decreased risk of death from all causes (95% CI 0.98 to 0.99).

**Conclusions:**

Women with postmenopausal status had a higher risk of all-cause mortality than premenopausal women, particularly for ages at menopause younger than 45 years.

**WHAT IS ALREADY KNOWN ON THIS TOPIC**

- Evidence of the impact on menopause on total mortality has been inconsistent, especially in China, where women’s reproductive characteristics are significantly different from those in Western women.

**WHAT THIS STUDY ADDS**

- Postmenopausal women had significantly increased risk of all-cause mortality compared with premenopausal women, particularly for women who had menopause at ages younger than 45 years.

**HOW THIS STUDY MIGHT AFFECT RESEARCH, PRACTICE OR POLICY**

- Our findings suggest that menopausal features may serve as significant mortality predictors for women at midlife for whom early monitoring and intervention strategies should be introduced to improve their long-term health.

## Introduction

 Menopause is defined as 12 months after a woman’s final menstrual period, marking the end of a woman’s reproductive life and permanent cessation of ovarian function.^1^ Most women typically experience menopause between their late 40s and early 50s globally.^2 3^ The mean age at menopause was 48 years in Chinese women, compared with over 50 years typically seen in Western populations.^4^,^6^ Menopause that occurs before the age of 40 years is defined as premature menopause,^7^ after age 40 but before age 45 years is known as early menopause.^7^ Through menopause, women experience a decline of sex hormones (eg, oestrogen and progesterone) secreted by the ovaries, and an increase in follicle stimulating hormone secreted by the pituitary and a decrease in anti-Mullerian hormone.^8^ A woman’s health may be impacted by the significant hormonal changes brought on by the menopausal transition.^9^ Previous research indicated that menopause may result in a large population at risk for long-term health consequences including mortality,^10^ with considerable public health impact.

In observational studies, the postmenopausal status coincided with an increased likelihood of developing multiple chronic conditions among women.^11^ Moreover, timing of onset of menopause matters to women’s health as well, since menopausal age has significant health implications. Age at menopause may be an important indicator not only of reproductive ageing but also of general health and somatic ageing.^12^ Numerous epidemiological studies have demonstrated that earlier or later menopause is linked to a variety of disease risks. Premature and early menopauses have been reported to be associated with greater risk of cardiovascular disease,^13^ type 2 diabetes,^14^ osteoporosis,^15^ fracture^16^ and worse cognitive function^17^ in later life. In contrast, late onset age of menopause has been associated with increased risk of cancers in the breast,^18^ endometrium^19^ and ovary.^20^

A few studies have investigated the relationship between age at menopause and all-cause mortality.^21^,^30^ However, most of the existing studies reported conflicting results. In addition, little is known about the association of menopause status (ie, premenopause, perimenopause and postmenopause) with women’s overall mortality. As previously reported, postmenopausal women showed a significant change in the concentration of plasma sex hormones (eg, oestrogen and progesterone depletion) compared with premenopausal women.^31^ At present, it still remains unclear regarding the effects of menopause features (status and age) on total mortality, particularly in China, where women’s reproductive characteristics are significantly different from those in Western women. In general, Chinese women tend to start their periods later, their menopause earlier and rarely use hormone replacement therapy. Moreover, the proportion of women who had a hysterectomy that include a prophylactic bilateral oophorectomy differs across countries.^32 33^ About 40% of women undergoing hysterectomy have had a prophylactic oophorectomy in the USA, whereas little is known about corresponding rates in Chinese women.^34^,^37^ Therefore, based on data from a prospective cohort study of the nationwide China Kadoorie Biobank (CKB), we examined the association of menopausal status and age at menopause with the risk of all-cause mortality including over 300 000 adult Chinese women.

## Materials and methods

### Study design and participants

**Figure 1:**
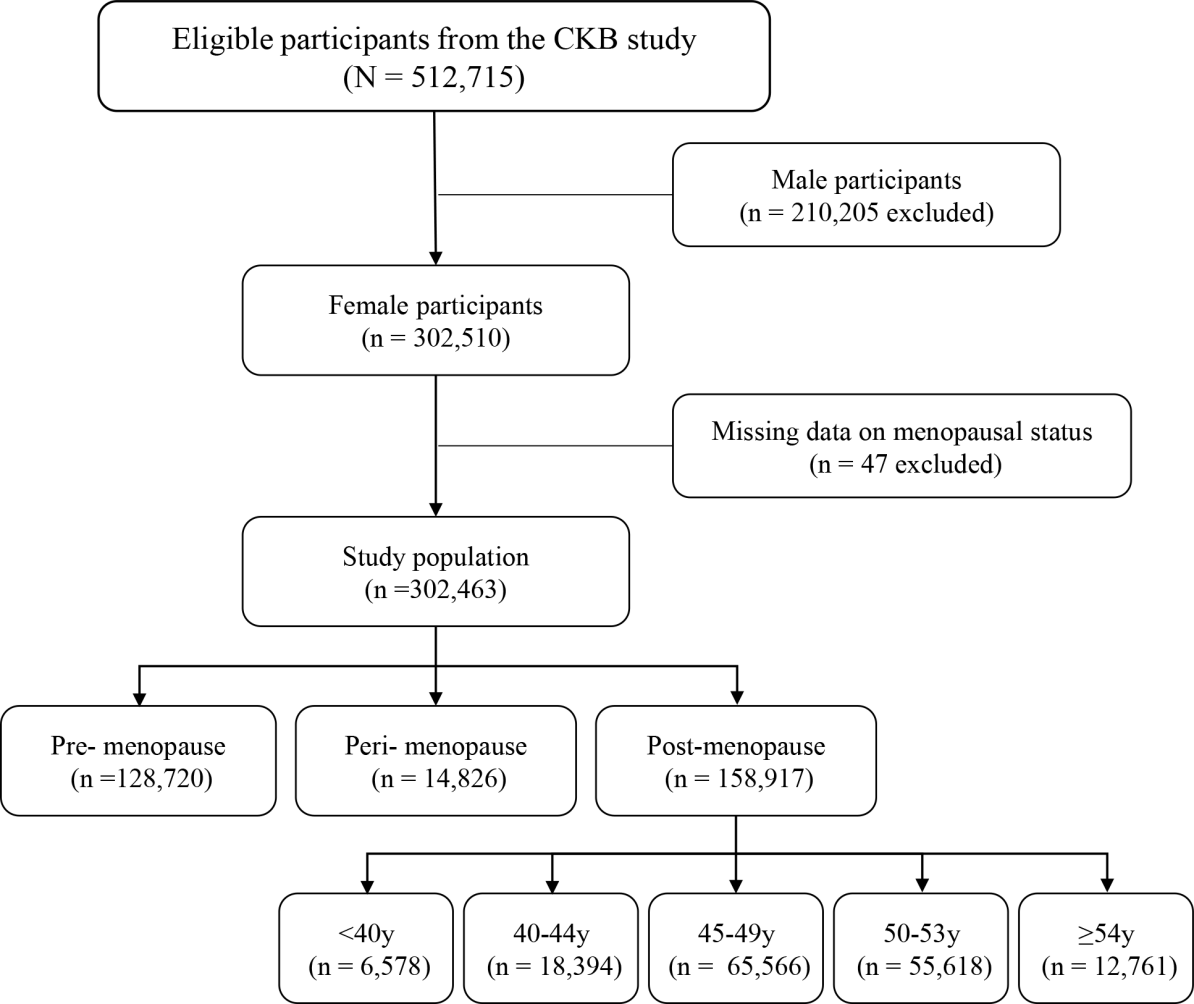
Flow diagram of inclusion or exclusion of study participants. CKB, China Kadoorie Biobank.

The CKB study is a sizeable prospective cohort study developed jointly by Oxford University and the Chinese Center for Disease Control and Prevention. Details on the CKB study design and methods have previously been extensively described elsewhere.^38 39^ Adults aged 30–79 years were recruited from five urban (Qingdao, Harbin, Haikou, Suzhou and Liuzhou) and five rural (Sichuan, Gansu, Henan, Zhejiang and Hunan) areas of China between 2004 and 2008. Selection of the survey sites was based on local patterns of disease and exposure to specific risk factors, population stability, quality of death and disease registries, local commitment, and capacity. In each administrative unit, all males and females who were permanently resident and without major disabilities were identified and invited to participate. The participation rate was ∼30%. A total of 512 715 participants (302 510 women) completed the baseline survey. Of these 302 510 female participants, 47 women who had missing data on menopausal status were excluded. The remaining 302 463 women were included in the present analyses (figure 1).

Data on sociodemographic characteristics, lifestyle factors, women’s reproductive history, personal and medical history (eg, coronary heart disease (CHD), stroke, hypertension, diabetes, chronic obstructive pulmonary disease (COPD) and cancer) were collected face-to-face using an interviewer-administered laptop-based questionnaire. Information on physical activity was obtained by asking participants about their usual type and duration of activities in occupational, commuting, housework and leisure-time related domains during the past 12 months. The metabolic equivalent task (MET, hours/day) value for a particular type of physical activity represents the ratio of the energy expended per kilogram of body weight per hour during that activity relative to that expended when sitting quietly. The number of hours spent per day participating in each activity was multiplied by the MET value for that activity, and the daily amount of total physical activity was calculated by summing the MET-hours/day for activities related to occupational, commuting, housework and leisure-time activities.^40^ Anthropometric measurements and physical measurements (eg, height and weight) were undertaken by trained health workers using calibrated instruments according to standardised protocols. Standing height was measured to the nearest 0.1 cm using a stadiometer. Weight was measured to the nearest 0.1 kg using the TBF-300GS Body Composition Analyser (Tanita, Tokyo, Japan), while participants were wearing light clothes (appropriate for the season) and no shoes. The reliability of anthropometric measurements was evaluated in a re-survey among a randomly chosen 5% of participants in 2008. The correlation coefficients between baseline and re-survey measures were quite high for height (0.99) and weight (0.96).^41 42^ Body mass index (BMI) was calculated as weight divided by the square of height (kg/m^2^).^38^

### Assessment of menopausal status and age at menopause

On the baseline questionnaire, women were asked ‘Have you had your menopause?’, with response options and relevant explanations as follows: (1) no (having regular menstrual cycle); (2) yes, currently (having irregular menstrual cycle but before 12 months of amenorrhoea); (3) yes, had menopause (having amenorrhoea for 12 months or more). The menopausal status of women was defined as premenopausal, perimenopausal or postmenopausal, when they reported that they had not, were currently or had menopause, respectively. In addition, women who had menopause were then further asked their age at completion of menopause. In categorical analysis of this study, the age at menopause was grouped as age less than 40 (premature menopause), 40–44 (early menopause), 45–49 (reference category), 50–53 and 54 years or older (later age at menopause). The reference group was consistent with the categories used in other epidemiological studies.^23 43^

### Follow-up for morbidity and mortality

Through connections to hospital records, national health registries and social health insurance databases in the research regions, data on deaths, including the causes of death, and health outcomes were periodically gathered from baseline to 31 December 2016. Active follow-up comprising community visits or direct contact with participants was carried out annually to minimise loss to follow-up.^38^ The 10th International Classification of Diseases was used as the criteria to code fatal occurrences recorded into the CKB follow-up system.^44^ All records, including scanned images of original death certificates, were reviewed centrally by study clinicians blinded to baseline information.^38^ Death from all causes was the outcome of this study’s analyses. Prospective follow-up on every participant was conducted, with a median follow-up of 10.20 years, and a retention rate of 100%.

### Statistical analysis

Baseline characteristics of the female participants were described as means (SD) for continuous variables and as counts (percentages) for categorical variables, according to menopausal status (premenopause, perimenopause and postmenopause) and age at menopause (<40, 40–44, 45–49, 50–53 and ≥54 years). Continuous variables were compared using the one-way analysis of variance test for variables with normal distribution and the Kruskal-Wallis test for variables with skewed distribution. Categorical variables were compared using the χ^2^ test. Missing values were treated and reported as missing in all analyses.

The analysis for menopausal status was conducted among all included women, while analysis for age at menopause was confined to postmenopausal women only, with the menopausal age at 45–49 years as the reference group. The outcome was divided into two categories by the presence of all-cause mortality (yes or no). Cox proportional hazards regression was used to estimate the HRs and 95% CIs for the associations between menopausal status, different categories of age at menopause and all-cause mortality. Moreover, we also calculated the HRs and 95% CIs for the risk of all-cause mortality associated with per additional year of age at menopause using Cox proportional hazards regression model. Based on prior knowledge, the statistical models were adjusted for the following potential confounders collected at baseline: age, region, BMI, level of highest education, annual household income, physical activity, smoking, alcohol consumption, age at menarche, parity, number of live births, age at first birth, breast feeding duration at first birth, CHD, stroke, hypertension, diabetes, COPD and cancer.

Subgroup analyses were also performed to obtain the adjusted HR and 95% CI for the association between per 1-year increase in age at menopause and all-cause mortality by age (30–39.9, 40–49.9, 50–59.9 or ≥60 years), BMI (<18.5, 18.5–23.9 or ≥24 kg/m^2^), study region (rural or urban), level of highest education (elementary school and below, middle and high school, or university and above), annual household income (<5000, 5000–19 999 or ≥20 000 Chinese yuan/year) (1 Chinese yuan was approximately equal to US$0.14), MET (<20, 20–29.9 or ≥30 hours/day), smoking (never or ever), alcohol consumption (never or ever), age at menarche (≤12, 13–14, 15–16 or ≥17 years) and parity (primiparous or multiparous). An interaction term was added into the model to assess the effect of age at menopause and baseline characteristics on the outcome of all-cause mortality. Statistical significance was set at p<0.05 for all statistical analyses and was performed with SAS V.9.4 (SAS Institute).

## Results

### Characteristics of study participants

Among the 302 463 women included, 42.56% of women were premenopausal, 4.90% perimenopausal and 52.54% postmenopausal at baseline (table 1). The mean age of premenopausal, perimenopausal and postmenopausal women was 41.98, 49.71 and 59.30 years, respectively. All participants had a mean age of 51.46 years, mean BMI of 23.82 kg/m^2^, mean MET values of 20.42 hours/day, mean age at menarche of 15.44 years, mean number of live births of 2.24, mean age at first birth of 23.38 years and mean breast feeding duration at first birth of 14.08 months. The majority of these women were rural residents (55.43%), had educational level of primary school and below (56.72%), had annual household incomes between 5000 and 19 999 yuan (49.16%) and were parous (98.63%). Few women ever smoked (5.06%) or drank alcohol (36.42%). Compared with premenopausal women, perimenopausal or postmenopausal women tended to have higher BMI, reside in urban regions, be less educated and less physically active, with higher prevalence of smoking, and have higher age at menarche, lower proportion of nulliparity, greater number of live births and longer breast feeding duration.

**Table 1:** Baseline characteristics of the study participants according to menopausal status and age at menopause (n=302 463)

Characteristic	Overall	Menopausal status			P value	Age at menopause					P value
		Premenopause	Perimenopause	Postmenopause		<40 years	40–44 years	45–49 years	50–53 years	≥54 years	
	N=302 463	N=128 720	N=14 826	N=158 917		N=6578	N=18 394	N=65 566	N=55 618	N=12 761	
Age (years), mean (SD)	51.46 (10.48)	41.98 (4.74)	49.71 (3.19)	59.30 (7.45)	<0.001	56.19 (10.91)	58.31 (8.88)	58.90 (7.46)	59.73 (6.47)	62.45 (5.47)	<0.001
BMI (kg/m^2^), mean (SD)	23.82 (3.46)	23.57 (3.23)	24.34 (3.35)	23.96 (3.63)	<0.001	23.83 (3.64)	23.63 (3.63)	23.82 (3.61)	24.14 (3.61)	24.48 (3.71)	<0.001
Missing	1	0	0	1		0	0	0	1	0	
Region, %					<0.001						<0.001
Rural	167 656 (55.43)	75 716 (58.82)	7574 (51.09)	84 366 (53.09)		3735 (56.78)	11 011 (59.86)	36 187 (55.19)	27 299 (49.08)	6134 (48.07)	
Urban	134 807 (44.57)	53 004 (41.18)	7252 (48.91)	74 551 (46.91)		2843 (43.22)	7383 (40.14)	29 379 (44.81)	28 319 (50.92)	6627 (51.93)	
Education, %					<0.001						<0.001
Elementary school and below	171 553 (56.72)	49 382 (38.36)	7773 (52.43)	114 398 (71.99)		4501 (68.43)	13 175 (71.63)	48 047 (73.28)	39 211 (70.50)	9464 (74.16)	
Middle and high school	117 474 (38.84)	70 925 (55.10)	6500 (43.84)	40 049 (25.20)		1904 (28.94)	4785 (26.01)	15 962 (24.34)	14 619 (26.28)	2779 (21.78)	
University and above	13 436 (4.44)	8413 (6.54)	553 (3.73)	4470 (2.81)		173 (2.63)	434 (2.36)	1557 (2.37)	1788 (3.21)	518 (4.06)	
Annual household income (yuan), %					<0.001						<0.001
Low (<5000)	30 715 (10.15)	7883 (6.12)	681 (4.59)	22 151 (13.94)		1087 (16.52)	3073 (16.71)	9149 (13.95)	6925 (12.45)	1917 (15.02)	
Middle (5000–19 999)	148 680 (49.16)	65 915 (51.21)	6913 (46.63)	75 852 (47.73)		3303 (50.21)	9291 (50.51)	31 700 (48.35)	25 720 (46.24)	5838 (45.75)	
High (≥20 000)	123 068 (40.69)	54 922 (42.67)	7232 (48.78)	60 914 (38.33)		2188 (33.26)	6030 (32.78)	24 717 (37.70)	22 973 (41.30)	5006 (39.23)	
Physical activity (MET hours/day), mean (SD)	20.42 (12.76)	24.71 (13.27)	21.59 (13.04)	16.83 (11.11)	<0.001	18.47 (12.48)	17.66 (11.69)	17.14 (11.27)	16.34 (10.70)	15.35 (10.11)	<0.001
Smoking, %					<0.001						<0.001
Never smoker	287 146 (94.94)	125 318 (97.36)	14 328 (96.64)	147 500 (92.82)		6069 (92.26)	16 853 (91.62)	60 843 (92.80)	51 877 (93.27)	11 858 (92.92)	
Ever smoker	15 317 (5.06)	3402 (2.64)	498 (3.36)	11 417 (7.18)		509 (7.74)	1541 (8.38)	4723 (7.20)	3741 (6.73)	903 (7.08)	
Alcohol consumption, %					<0.001						<0.001
Never drinker	192 298 (63.58)	76 274 (59.26)	8712 (58.76)	107 312 (67.53)		4326 (65.76)	12 409 (67.46)	43 955 (67.04)	37 909 (68.16)	8713 (68.28)	
Ever drinker	110 165 (36.42)	52 446 (40.74)	6114 (41.24)	51 605 (32.47)		2252 (34.24)	5985 (32.54)	21 611 (32.96)	17 709 (31.84)	4048 (31.72)	
Age at menarche (years), mean (SD)	15.44 (1.97)	14.87 (1.75)	15.35 (1.91)	15.92 (2.02)	<0.001	15.64 (2.45)	15.67 (2.10)	15.91 (1.97)	15.97 (1.98)	16.24 (2.05)	<0.001
Parity, %					<0.001						<0.001
Nulliparous	4142 (1.37)	1916 (1.49)	138 (0.94)	2088 (1.31)		319 (4.85)	299 (1.63)	809 (1.23)	541 (0.97)	120 (0.94)	
Parous	298 321 (98.63)	126 804 (98.51)	14 688 (99.06)	156 829 (98.69)		6259 (95.15)	18 095 (98.37)	64 757 (98.77)	55 077 (99.03)	12 641 (99.06)	
Number of live births, mean (SD)	2.24 (1.34)	1.58 (0.79)	1.75 (0.88)	2.81 (1.47)	<0.001	2.65 (1.62)	2.87 (1.62)	2.78 (1.46)	2.76 (1.40)	3.15 (1.44)	<0.001
Missing	2881	1211	98	1572		262	226	602	394	88	
Age at first birth (years), mean (SD)	23.38 (3.19)	23.71 (2.80)	24.28 (2.94)	23.03 (3.46)	<0.001	22.71 (3.24)	22.83 (3.39)	23.04 (3.39)	23.19 (3.58)	22.74 (3.49)	<0.001
Missing	4142	1916	138	2088		317	294	797	528	115	
Breast feeding duration at first birth (months), mean (SD)	14.08 (8.22)	13.09 (7.39)	14.12 (8.68)	14.87 (8.72)	<0.001	14.82 (9.35)	15.10 (9.20)	15.06 (8.90)	14.53 (8.23)	15.05 (8.76)	<0.001
Missing	4142	1916	138	2088		317	294	797	528	115	

BMIbody mass indexMETmetabolic equivalent of activities

Among the 158 917 postmenopausal women at baseline, the mean age at menopause was 48.21 years, 6578 (4.14%) experienced menopause before the age of 40 years, 18 394 (11.57%) experienced menopause at 40–44 years, 65 566 (41.26%) experienced menopause at 45–49 years, 55 618 (35.0%) experienced menopause at 50–53 years and 12 761 (8.03%) experienced menopause at 54 years or above (table 1). Compared with women who had later menopause at baseline, those had earlier menopause were, on average, younger, more likely to have lower mean of BMI, be resident in rural areas, less educated, have lower household income, be more active, smoke more, have had lower age at menarche and with a higher proportion of nulliparity.

### Menopausal status, age at menopause and risk of all-cause mortality

**Figure 2:**
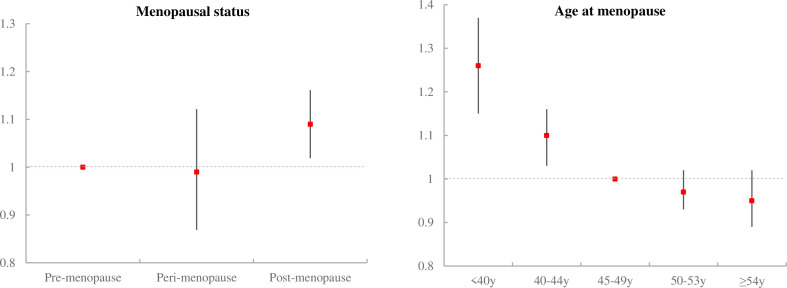
Cox proportional HRs (95% CIs) for all-cause mortality, according to menopausal status and age at menopause. Analyses were adjusted for age, region, body mass index, level of highest education, annual household income, physical activity, smoking, alcohol consumption, age at menarche, parity, number of live births, age at first birth, breast feeding duration at first birth, coronary heart disease, stroke, hypertension, diabetes, chronic obstructive pulmonary disease and cancer.

Compared with premenopausal women, postmenopausal women had statistically significantly higher risk of all-cause mortality, with adjusted HR of 1.09 (95% CI 1.02 to 1.16) (table 2, figure 2). However, the associations were not significant in perimenopausal women after adjusting for potential confounders (adjusted HR: 0.99, 95% CI 0.87 to 1.12).

**Table 2:** Cox proportional HRs (95% CIs) for all-cause mortality, according to menopausal status and age at menopause

	Number of women	Number of deaths	Model 1	Model 2
			Crude HR (95% CI)	Adjusted HR (95% CI)
Menopausal status				
Premenopause	128 720	1442	1.00 (reference)	1.00 (reference)
Perimenopause	14 826	299	1.81 (1.60 to 2.05)*	0.99 (0.87 to 1.12)
Postmenopause	158 917	11 882	6.91 (6.54 **to** 7.30)*	1.09 (1.02 **to** 1.16)*
Age at menopause (years)				
<40	6578	603	1.28 (1.18 to 1.39)*	1.26 (1.15 to 1.37)*
40–44	18 394	1583	1.20 (1.13 to 1.27)*	1.10 (1.03 to 1.16)*
45–49	65 566	4749	1.00 (reference)	1.00 (reference)
50–53	55 618	3867	0.96 (0.92 to 1.001)	0.97 (0.93 to 1.02)
≥54	12 761	1080	1.18 (1.10 to 1.26)*	0.95 (0.89 to 1.02)
Per additional year			0.99 (0.98 to 0.99)*	0.99 (0.98 to 0.99)*

Model 1: unadjusted.

Model 2: adjusted for age, region, body mass index, level of highest education, annual household income, physical activity, smoking, alcohol consumption, age at menarche, parity, number of live births, age at first birth, breast feeding duration at first birth, coronary heart disease, stroke, hypertension, diabetes, chronic obstructive pulmonary disease and cancer.

* p<0.01.

CIconfidence intervalHRhazard ratio

Among women who had undergone menopause, women with a lower age at menopause had a significantly higher risk of overall mortality, with adjusted HR of 1.26 (95% CI 1.15 to 1.37) for premature menopause (<40 years) and 1.10 (95% CI 1.03 to 1.16) for early menopause (40–44 years), relative to 45–49 years. However, women with menopause at later age were not significantly associated with risk of all-cause mortality after adjustments (adjusted HR: 0.97, 95% CI 0.93 to 1.02 for women who had menopause at age 50–53 years; adjusted HR: 0.95, 95% CI 0.89 to 1.02 for women who had menopause at aged ≥54 years).

In addition, a statistically significant inverse association was found between each year of delay of age at menopause and risk of total mortality. A 1-year increase in the age at menopause was associated with 1% reduction in all-cause mortality (95% CI 0.98 to 0.99).

### Age at menopause and risk of all-cause mortality, stratified by baseline characteristics

Although the magnitude of the associations varied slightly, the above-described associations between age at menopause and total mortality were broadly consistent across most subgroups of women, including age, BMI, education, household income, MET, smoking, alcohol consumption and parity (all p for heterogeneity >0.05) (table 3).

**Table 3:** Cox proportional HRs (95% CIs) for all-cause mortality per year increase in age at menopause, stratified by baseline characteristics, among postmenopausal women only

	Number of deaths	Adjusted HR (95% CI)	P value	P for heterogeneity
Age (years)				0.38
30–39.9	398	1.11 (0.93 to 1.33)	0.24	
40–49.9	1345	1.003 (0.97 to 1.03)	0.84	
50–59.9	3328	0.99 (0.98 to 0.999)*	0.045	
≥60	8552	0.98 (0.98 to 0.99)†	<0.001	
BMI (kg/m^2^)				0.22
<18.5	1336	0.99 (0.98 to 1.001)	0.08	
18.5–23.9	6450	0.98 (0.98 to 0.99)†	<0.001	
≥24	5837	0.99 (0.98 to 0.995)†	<0.001	
Region				0.02
Rural	8693	0.98 (0.98 to 0.99)†	<0.001	
Urban	4930	0.99 (0.99 to 1.001)	0.09	
Education				0.08
Elementary school and below	10 928	0.99 (0.98 to 0.99)†	<0.001	
Middle and high school	2465	0.997 (0.99 to 1.008)	0.57	
University and above	230	0.97 (0.94 to 1.008)	0.13	
Annual household income (yuan)				0.15
<5000	3276	0.98 (0.97 to 0.99)†	<0.001	
5000–19 999	7013	0.99 (0.98 to 0.99)†	<0.001	
≥20 000	3334	0.99 (0.98 to 1.001)	0.09	
MET (hours/day)				0.71
<20	10 654	0.99 (0.98 to 0.99)†	<0.001	
20–29.9	1650	0.99 (0.97 to 0.998)*	0.02	
≥30	1319	0.98 (0.97 to 0.999)*	0.03	
Smoking				0.11
Never smoker	11 937	0.99 (0.98 to 0.99)†	<0.001	
Ever smoker	1686	0.99 (0.98 to 1.004)	0.19	
Alcohol consumption				0.68
Never drinker	9690	0.99 (0.98 to 0.99)†	<0.001	
Ever drinker	3933	0.99 (0.98 to 0.994)†	<0.001	
Age at menarche (years)				0.04
≤12	544	1.004 (0.98 to 1.02)	0.73	
13–14	2900	0.99 (0.98 to 1.001)	0.08	
15–16	5077	0.99 (0.98 to 0.99)†	<0.001	
≥17	5102	0.98 (0.98 to 0.99)†	<0.001	
Parity				0.32
Nulliparous	253	0.92 (0.80 to1.06)	0.23	
Parous	13 370	0.99 (0.98 to 0.99)†	<0.001	

Adjustments are as in table 2 model 2, except for the stratified variable in the corresponding stratified analysis.

* p<0.05.

† p<0.01.

BMIbody mass indexMETmetabolic equivalent of activities

**Figure 3:**
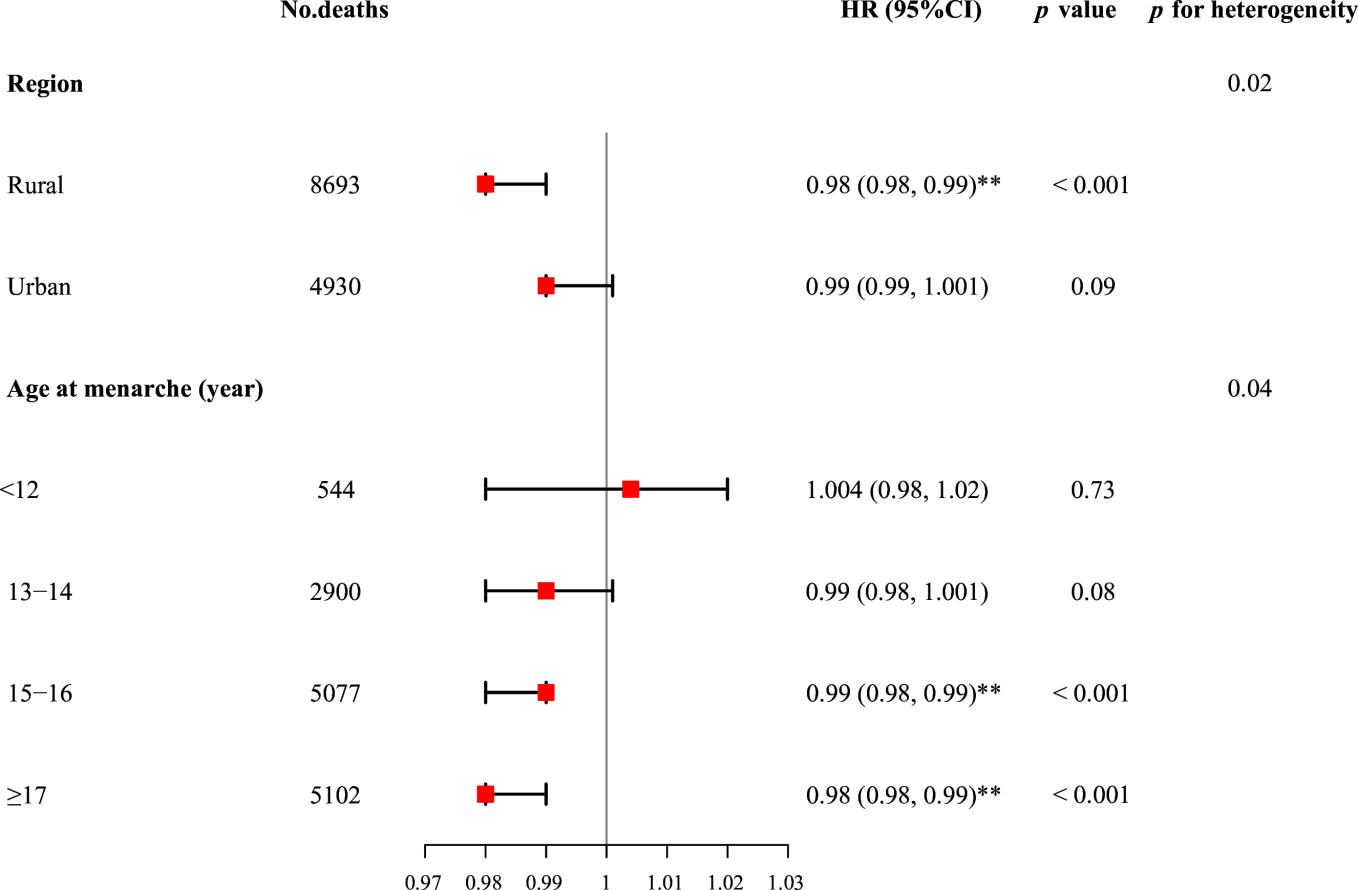
Cox proportional HRs (95% CIs) for all-cause mortality per year increase in age at menopause, stratified by baseline characteristics, among postmenopausal women only. Adjustments are as in table 2 model 2, except for the stratified variable in the corresponding stratified analysis. **p<0.01, *p<0.05.

However, the inverse relationship of increasing age at menopause with all-cause mortality was more pronounced among women who resided in rural areas (adjusted HR: 0.98, 95% CI 0.98 to 0.99) than urban area (adjusted HR: 0.99, 95% CI 0.99 to 1.001) (p for heterogeneity=0.02), and whose age at menarche was 15–16 years (adjusted HR: 0.99, 95% CI 0.98 to 0.99) and ≥17 years (adjusted HR: 0.98, 95% CI 0.98 to 0.99) compared with ≤12 years (adjusted HR: 1.004, 95% CI 0.98 to 1.02) and 13–14 years (adjusted HR: 0.99, 95% CI 0.98 to 1.001) (p for heterogeneity=0.04) (table 3 and figure 3).

## Discussion

Based on a large prospective cohort study of Chinese women, we found that postmenopausal women had significantly increased risk of all-cause mortality compared with premenopausal women after adjusting for multiple confounding variables, including age. Furthermore, among postmenopausal women, premature menopause (<40 years) or early menopause (40–44 years) was associated with higher risk of all-cause death, and a 1-year delay in menopause was associated with a 1% reduction in the risk of all-cause mortality. Nevertheless, our study did not observe statistically significant associations for later menopause with all-cause mortality. To our knowledge, this is the first and largest prospective study in mainland China to investigate the associations of menopausal status and age at menopause with overall mortality risk.

The menopause transition affects women’s health uniquely and in various ways, and accumulating evidence have focused on the effects of the menopausal transition and postmenopause stages on the risk of certain health problems.^43 45^ However, to date, despite the observed occurrence of several crucial metabolic and cardiovascular disease risks during the menopause transition,^46^ we have not identified any published studies that have analysed the association between specific premenopausal, perimenopausal or postmenopausal status and women’s mortality, and it is not clear what role menopause transition plays in a woman’s risk of total mortality across population-based studies. According to prior studies, several biological mechanisms have been postulated to explain the effect of menopausal status on mortality, and the most promising interpretation may be the loss of endogenous oestrogens during the menopause transition and postmenopausal period.^47^,^49^ As the primary female sex hormone, oestrogens play an important role in the regulation of complex physiological processes, ageing and multiple disease states.^50^ Fluctuations in the oestrogen hormone levels and decline in oestrogens during the menopausal transition have been shown to drive a systemic inflammatory state, which can potentiate immune and metabolic dysfunction, as well as the development of a variety of major chronic conditions,^51^ and therefore significantly contributing to an increased risk of premature death in postmenopausal women.^52^ Furthermore, oestrogen deficiency caused by premature or early menopause has been implicated in tissue or organ dysfunctions and lesions via hormonal changes, which are closely linked with long-term health risks including increased overall mortality.^10^ Further research is needed to investigate the related mechanisms by which the menopause transition and menopause at an early age may affect the risk of all-cause mortality, and thus prevent menopause-related adverse effects.

Moreover, in the analysis of postmenopausal women, after adjusting for multiple potential confounders, including demographic, socioeconomic, lifestyle, other reproductive factors and chronic medical conditions, significantly increased risk of all-cause mortality was observed for early onset of menopause, which is in line with results from most of the previous studies.^21^,^28^ In a 12-year follow-up of 6182 California Seventh-Day Adventist women, there was a 30% increase in all-cause mortality for women with a natural menopause at <40 years of age.^27^ Similar results of total mortality risk were observed in a large population of postmenopausal women with menopause at age 40–44 years,^28^ and <45 years^22^ based on a follow-up study in the USA. Another cohort study of 19 731 Norwegian postmenopausal women with 37 years of follow-up also demonstrated an inverse relation between the age at menopause and all-cause mortality risk, showing that a 3-year increase in the age at menopause was associated with 1.6% reduction in total mortality.^23^ Similarly, a cohort study with approximately 16 years of follow-up of 2658 South Korean women found that natural menopause before the age of 40 years had a 32% higher all-cause mortality.^26^ In two cohort analyses of Chinese postmenopausal women from Taiwan and Shanghai, authors found that experiencing menopause at the age of 45–49 years,^24^ or before the age of 46.64 years^21^ was also associated with higher risk of total mortality. More recently, in a meta-analysis of 16 studies involving 321 233 women, the results also revealed a statistically significant association of natural menopause age under 40 years with all-cause mortality risk.^25^

In addition, our study did not observe statistically significant associations for late age at menopause and total mortality after controlling for confounding factors. However, several studies have yielded contradictory findings, showing that women with later menopause were at a decreased risk of all-cause mortality.^29 53^ This discrepancy may be possibly attributed to differences in participant characteristics, study design, sample sizes and age range at menopause. Notably, our study revealed that per 1-year delay in menopause was related to a 1% reduction in the risk of all-cause mortality, and the associations tended to be more pronounced among women who lived in rural areas, and with older age at menarche. The reasons for these pronounced inverse associations across different population subgroups were not clear, and additional prospective studies are warranted.

The major strengths of the present study, including the prospective design, large sample size and diverse geographic regions covered, contributed to the generalisation of study findings to the general population in China. In addition, the high quality and completeness of data collection, stringent case ascertainment via comprehensive follow-up systems, and the comprehensive adjustment for covariables simultaneously limit possible confounding bias in the analyses. However, this study had several limitations. First, the information of menopause was self-reported by the participants and may, therefore, be subject to recall bias. Second, in the self-reported questionnaire, the cause for menopause, menopausal symptoms, female hormones, use of hormone therapy and hyperlipidaemia were not collected, which may have influenced the observed association between menopause characteristics and death from all causes. However, previous studies have indicated that early menopause whether natural or induced was associated with increased risk of all-cause mortality.^10^ Third, our analysis only established an association between early menopause and all-cause mortality, thus further investigations of the underlying biological mechanisms are warranted. Fourth, the present study was also limited by the lack of information on hysterectomy and oophorectomy, future research should consider the potential effects of these factors on the observed association. In addition, despite allowance for a comprehensive set of potential confounders, residual confounding from unmeasured or other potential risk factors may still exist given the observational nature of this study.

## Conclusion

In conclusion, our large-scale longitudinal study provided robust evidence that women had significantly higher risk of total mortality during postmenopause. In particular, earlier age at menopause was associated with increased risk of all-cause mortality. Our findings indicated that menopausal features may serve as significant mortality predictors for women at midlife when early monitoring and intervention strategies should be introduced to improve their long-term health. Further studies are needed to confirm these findings and to clarify the mechanisms that link menopause characteristics to mortality risk.

## Data Availability

Data are available upon reasonable request.
